# Environmental Life Cycle Assessment of the Materials, Components, and Elements of a Mono-Si Photovoltaic Power Plant

**DOI:** 10.3390/ma18122748

**Published:** 2025-06-11

**Authors:** Patryk Leda, Izabela Piasecka, Grzegorz Szala

**Affiliations:** 1 Faculty of Mechanical Engineering, Faculty of Mechatronics, Kazimierz Wielki University, Mikołaja Kopernika 1, 85-074 Bydgoszcz, Poland; 2 Faculty of Mechanical Engineering, Bydgoszcz University of Science and Technology, Al. Prof. S. Kaliskiego 7, 85-796 Bydgoszcz, Poland; izabela.piasecka@pbs.edu.pl; 3 Faculty of Mechatronics, Kazimierz Wielki University, Mikołaja Kopernika 1, 85-074 Bydgoszcz, Poland; gszala@ukw.edu.pl

**Keywords:** Life Cycle Assessment (LCA), photovoltaic power plant, ReCiPe 2016, renewable energy sources, sustainable development

## Abstract

The main objective of this study is to assess the environmental life cycle of the materials, components, and elements of a mono-Si photovoltaic power plant towards their sustainable development. Currently, photovoltaic installations are considered to be environmentally friendly systems that produce “green” energy. During their exploitation, no pollutants are emitted into the environment. However, the processes of manufacturing and post-used management of their materials, components and elements are associated with both high demand for energy and matter, as well as with emissions of harmful substances into the atmosphere, water, and soil. For this reason, from the perspective of the entire life cycle, photovoltaic power plants may contribute to the deterioration of human health, the reduction in the quality of the environment, and the depletion of non-renewable fossil resources. Due to these potential threats, it was considered appropriate to conduct a Life Cycle Assessment of a real 2 MW photovoltaic power plant located in northern Poland, in terms of compliance with the main assumptions of sustainable development. The analysis was conducted using the Life Cycle Assessment (LCA) methodology (the ReCiPe 2016 model). Impacts on the environment was assessed in three areas: human health, ecosystem quality, and material resources. Two scenarios were adopted for the post-used management of materials, components, and elements: landfill disposal and recycling. Based on the conducted research, it was found that, among the assessed groups of photovoltaic power plant components (photovoltaic modules, supporting structure, inverter station, and electrical infra-structure), photovoltaic modules have the highest level of harmful impact on the environment (especially the manufacturing stage). The use of recycling processes at the end of their use would reduce their harmful impact over the entire life cycle of a photovoltaic power plant and better fit with the main principles of sustainable development.

## 1. Introduction

### 1.1. Background

Sustainable development is an aspect of intergenerational solidarity focused on developing solutions that ensure future progress, allowing for all social groups to actively participate in development processes while also benefiting from economic prosperity [1].

Initially, conversations about sustainable development focused on the need to lessen economies’ detrimental influence on the natural environment. The notion has evolved over time, including three key development factors: environmental stewardship, social improvement, and economic prosperity. The notion of sustainable development is currently gaining traction in socioeconomic development discussions, becoming a horizontal principle mirrored in worldwide development plans [1].

The UN 2030 Agenda for Sustainable Development consists of 17 sustainable development objectives and 169 associated targets. Goal 7 calls for guaranteeing access to cheap, dependable, sustainable, and modern energy. Countries worldwide are still attempting to achieve sustainable energy targets, but their rate of progress is too slow. If present trends continue, over 660 million people will remain without access to power by 2030, and nearly 2 billion people will be compelled to prepare their meals using harmful fuels and technologies. Renewable energy sources now account for about 30% of total energy consumption in the electricity sector, while their proportion in heating and transportation remains quite low. Renewable energy installations in poor nations increase by 9.6% per year; however, despite rising demand, international funding flows for “clean” energy continue to shrink [2,3].

The percentage of the population with access to electricity has risen from 73% in 1998 to 91% by 2024. However, 675 million people globally (mainly in the least-developed nations and Sub-Saharan Africa) continue to live without access to electricity, accounting for almost one in every ten people. To attain universal energy access by 2030, we must expedite electrification, expand investments in renewable energy sources, enhance energy efficiency, and create enabling policies and regulatory frameworks. Everyday living depends on reliable and inexpensive energy. At the same time, energy consumption remains a significant contributor to climate change, accounting for over 60% of worldwide greenhouse gas emissions [2,4].

**Figure 1:**
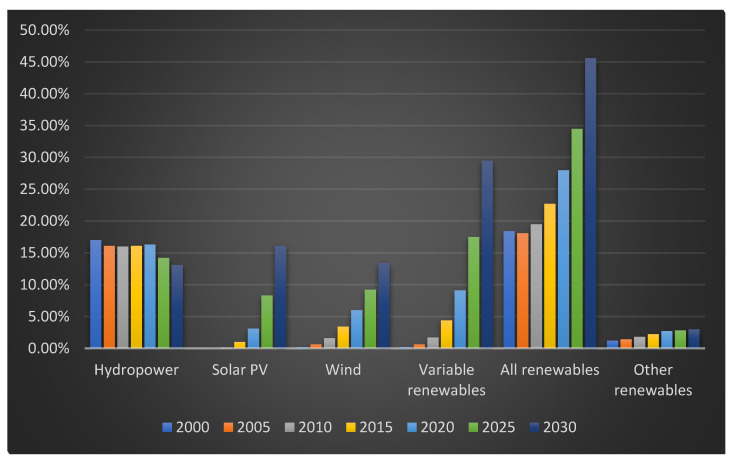
Share of renewable electricity generation by technology, 2000–2030, Own work based on [5].

By the end of the decade, worldwide renewable energy output is predicted to surpass 17,000 TWh, representing an almost 90% increase from 2023. The renewable energy market is predicted to grow significantly during the next five years (Figure 1). By the end of 2025, renewable energy generation is predicted to outperform coal. By a year later, in 2026, wind and solar power are predicted to outperform nuclear energy. The next significant breakthrough is predicted to come in 2029, when photovoltaic electricity will overtake hydropower as the world’s top renewable energy source. By 2030, wind power is predicted to outperform hydropower [5].

In accordance with the above factors, it was considered reasonable to assess the environmental life cycle of materials, components, and elements of a mono-Si photovoltaic power plant towards their sustainable development.

### 1.2. Literature Review

LCA studies in Poland remain less popular than in other nations across the world. However, this is a topic that will be widely utilized in the evaluation of photovoltaic power plants in a few or a dozen years, when the operational term comes to an end.

So far, there have been several studies conducted just on photovoltaic modules. As a result, existing research is limited to photovoltaic panels. This research does not account for all of the components of a photovoltaic power plant, including its photovoltaic panels, support structures, electrical systems, and inverter stations.

Several studies in the worldwide literature have used ReCiPe 2016 technology to undertake LCAs for photovoltaic power plant projects. However, most studies focus on the influence of photovoltaic panel life cycles on global warming potential (GWP), neglecting additional negative consequences for the ecosystem, the environment surrounding humans, human health, and material resources that degrade. Recent investigations by other experts have concentrated on photovoltaic panels rather than entire PV power facilities.

Life Cycle Assessment (LCA) is critical for determining the environmental effect of photovoltaic systems. Recent research has focused on several PV technologies, such as bifacial panels, which are predicted to dominate the industry by 2030 (Maniscalco et al. [6]). LCA research considers energy indices such as energy payback time and cumulative energy demand, as well as environmental implications; however, techniques and impact assessment methods differ (Muteri et al. [7]). PV type, geographical location, and system performance are all significant influences on LCA findings (Muteri et al. [7]). Material selection influences the environmental performance of PV systems (Portillo et al. [8]). While LCA is useful for micro-scale study of goods and generating systems, it has limits for large-scale evaluations (Ito [9]). To enhance the assessment of renewable energy systems, LCA approaches must be standardized and environmental, economic, and social issues must be integrated (Portillo et al. [8]).

Was also investigated organic and perovskite photovoltaic cells with transparent graphene electrodes [10], while Li et al. revisited organic and perovskite photovoltaic cells [11]. Piasecka et al. [12] and Mao et al. explored crystalline silicon photovoltaic panels [13], Li et al. investigated flexible photovoltaic cells [14], Elnozahy et al. investigated photovoltaic panel energy efficiency [15], and Li et al. examined some panels [16]. Muteri et al. [7], Ren et al. [17], and Ludin et al. [18] conducted studies on the overall impact of photovoltaic panels.

Other studies have investigated the total environmental impact of photovoltaic systems [19,20,21,22,23,24,25,26,27,28,29,30]. Various studies focus on diverse regions, including Pakistan [31], New York [32], and Texas [33].

Therefore, in the world literature on the Life Cycle Assessment of photovoltaic systems, there are no studies in which analyses would be performed using the ReCiPe 2016 method and would focus not only on the assessment of the level of greenhouse gas emissions, but also on the impact of individual construction materials components, and elements on human health, environmental quality, and the depletion of non-renewable raw materials. Currently, most photovoltaic power plants are based on panels made of monocrystalline silicon. Their post-used management after the end of exploitation will pose a huge challenge in the future, both environmentally and economically.

### 1.3. Research Contribution

Our findings allow us to highlight the environmental effects of the manufacturing, usage, and post-consumer treatment of used solar panels and other photovoltaic power plant components. This field of study requires ongoing development and implementation.

Sustainable management of the life cycle of technological renewable energy facilities is made feasible primarily by conducting environmental studies of the cycle’s activities. At the same time, in addition to environmental models, economic assessments are required to determine the viability of the investment, as unprofitable systems have little chance of becoming popular in society. However, if society has a significant aversion to a specific sort of technological capability (in the case of RES, this is generally due to ignorance), the odds of popularizing that solution are slim.

The Earth is made up of interrelated ecosystems that have an impact on human health, either directly or indirectly. As a result, the primary study goal of this article is to assess the environmental life cycle of materials, components, and elements of a mono-Si photovoltaic power plant towards their sustainable development.

Sustainable development in renewable energy systems requires minimizing negative impacts, maximizing positive impacts, reducing costs, increasing economic benefits, and educating society about renewable energy sources.

The publication process began with a review of the literature on the subject of research. We provided grounds for pursuing the issue specified in the title, which also includes the key research problem. The suitability of the analysis selection was assessed utilizing the LCA (Life Cycle Assessment) approach. Section LCI allowed for a thorough investigation of the structure of the tested solar power plant, including the percentage contribution of different parts and materials. Relevant simulation analyses were carried out using the SimaPro 9.4.0 software and the ReCiPe 2016 calculation procedure. This stage’s evolution is documented. This inquiry concluded with an interpretation of the obtained results.

## 2. Materials and Methods

### 2.1. Object of Analysis

**Figure 2:**
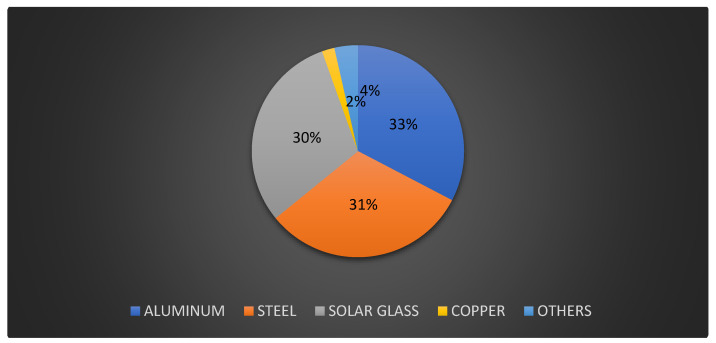
Percentage distribution of component mass in the tested photovoltaic power plant (investor data).

**Figure 3:**
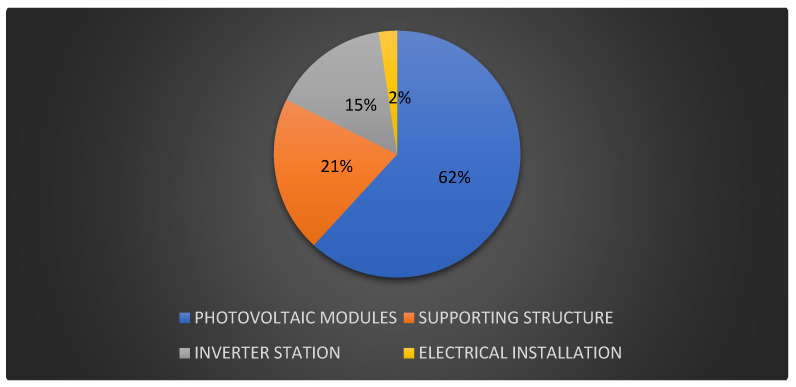
Percentage division of the mass of materials of the considered photovoltaic power plant (investor data).

**Figure 4:**
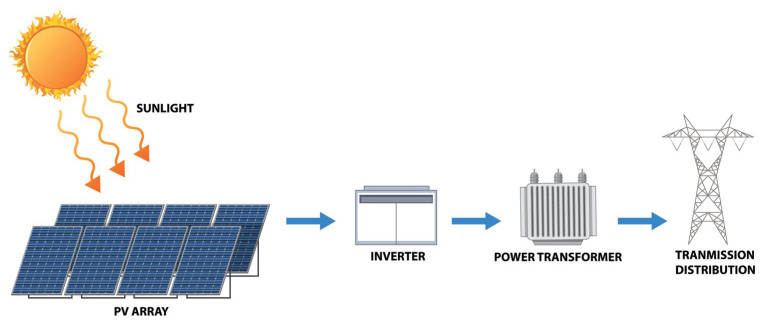
Block diagram of the identified photovoltaic power [34].

This research focuses on a 2 MW photovoltaic power plant in northern Poland. This power plant can generate around 1900–2200 MWh per year (figures based on 8 years of operation). Because of variable weather conditions, the average value of produced power may vary by around 10%. As a result, this research estimates that the power plant generates an average of 2000 MWh each year. The combined mass of materials, supplies, and components in the tested photovoltaic power plant is approximately 300,000 kg (Figure 2). Figure 3 reveals the list of photovoltaic power plant elements. The photovoltaic panels face south at a 40-degree slant. The tested power plant has 8334 polycrystalline photovoltaic modules with a capacity of 240 W each. A block diagram of the identified photovoltaic power plant is also displayed in Figure 4.

### 2.2. Methodology

LCA (Life Cycle Assessment) is a way of determining a product’s possible environmental impacts. It entails examining the quantity of raw materials, energy, and waste produced before determining their influence on the natural environment, human health, and raw material supplies. This process encompasses a product’s whole life cycle, beginning with the acquisition of raw materials and progressing through manufacturing and distribution to disposal. Using LCA in the design of renewable energy technologies enables the effective management of materials and energy at all stages of their life, resulting in lower raw material consumption and a larger proportion of recycling [35,36,37].

The comprehensive method of LCA is currently mostly implemented using the following international standards:

(a) EN ISO 14040 Environmental management—life cycle assessment—principles and framework [38];
(b) EN ISO 14044. Environmental management—Life cycle assessment—Requirements and guidelines [39].

According to the standards, Life Cycle Assessment (LCA) considers the environmental elements and possible consequences on the environment and ecosystems throughout the product’s life cycle, from raw material acquisition to manufacturing, consumption, processing, recycling, or landfill [38,39].

**Figure 5:**
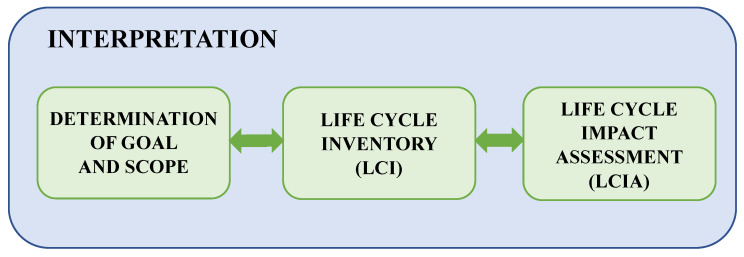
A diagram demonstrating the main processes used in LCA analysis [26,30].

The life cycle research includes four phases: establishing the purpose and scope, inventory analysis, impact assessment, and interpretation [26,29,38,39,40] (Figure 5).

The Life Cycle Assessment (LCA) approach evaluates possible environmental and ecological hazards. Their identification is accomplished by estimating the amount of materials used and energy demand in manufacturing processes, as well as the amount of waste generated and released into the environment, and then analyzing the impact of these processes on the quality of the natural environment, human health, and raw material depletion [26,27,28,40].

Analyses encompass the full product life cycle, beginning with the extraction of raw materials required for manufacturing and progressing through the manufacturing and distribution procedures for post-use management. This means that adopting the LCA technique to develop technical facilities for renewable energy allows for the more effective management of matter and energy throughout their life cycles [26,27,28,30,41].

The ReCiPe 2016 approach was employed to perform this research. The simulation was run using SimaPro 9.4 software.

ReCiPe 2016 is an approach with the broadest collection of effect categories compared to prior models. ReCiPe 2016 is an upgrade on ReCiPe 2008 and previous variants such as Eco-indicator 99. Unlike the previous edition, ReCiPe 2016 considers both local and global issues, making it ideal for analyzing the availability cycle of technical infrastructure for renewable energy [26,29,40].

As a result, the ReCiPe 2016 technique was chosen to assess the impact of materials, components, and elements of the photovoltaic power plant on the environment, human health, and raw material resources.

The LCA technique concludes with the interpretation of the acquired results. The interpretation step provides the foundation for forming the findings and summarizing judgments taken in-line with the previously specified purpose and scope.

## 3. Results

The ReCiPe 2016 model’s findings are reported for both the overall photovoltaic power plant and its various groupings of elements (support structure, PV panels, inverter station, and electrical installation). There were two distinct possibilities for the post-used management of plastics, materials, components, and functioning units: landfilling (to the maximum degree feasible) and recycling methods. Three categories of influence were assessed: human health, ecosystems, and raw material depletion. Their overall environmental effect was compared, and each was thoroughly examined, demonstrating the magnitude of the most important material emissions and the influence of the most essential processes. Furthermore, the possible environmental effect of the life cycles of the most essential materials used in the construction of the power plant’s parts and functioning units was provided.

The grouping and weighting results are reported as environmental points (Pt). One thousand environmental points represent an average European resident’s environmental contribution over the course of a year.

### 3.1. Total Impact

Table 1 summarizes the results of grouping and weighting the environmental consequences of the analyzed photovoltaic power plant’s life cycle in terms of the effects of emissions on human health, the ecosystem, and raw material resources, while accounting for the areas of impact (ReCiPe 2016 model). Two possibilities for the post-used management of plastics, materials, and components were considered: landfilling and recycling. The impact on human health (8.30 × 10^5^ Pt life cycle with storage management) had the most severe environmental repercussions. Recycling had the most beneficial impact on human health (−2.39 × 10^5^ Pt) of any post-used management method. Recycling as a kind of post-used management would lower the quantity of hazardous emissions produced throughout the life cycle of the tested technological facility.

**Figure 6:**
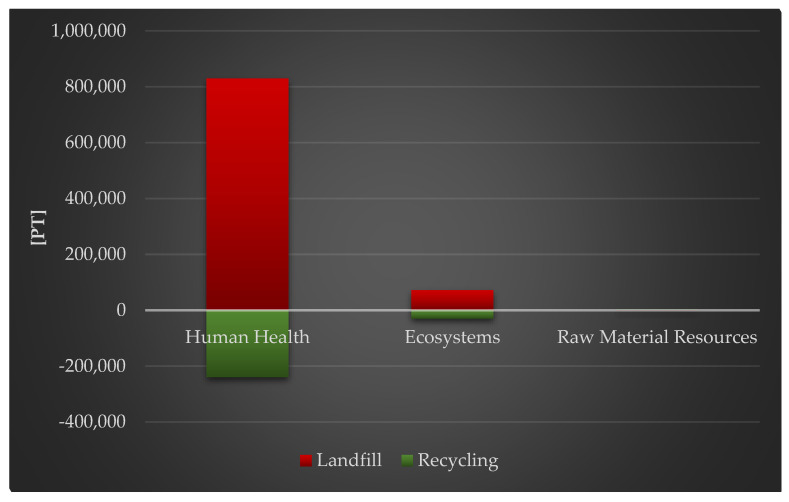
Grouping and weighting the total consequences for the environment of the life cycle of the analyzed photovoltaic power plant, regarding their impact on human health, the ecosystem, and raw material resources (ReCiPe 2016 model), taking into account the method of the post-used management of materials, components, and elements [unit: Pt].

Figure 6 depicts the entire effect values of a photovoltaic power plant’s life cycle, taking into consideration post-used management methods (landfill, recycling) and impact areas (human health, ecology, raw material resources). Recycling has a crucial role in reducing negative environmental repercussions, both in terms of human health and ecological quality.

Table 2 describes the findings of grouping and weighting the effects on human health, the ecology, and raw material resources caused by the life cycle of individual photovoltaic power plant units. The ReCiPe 2016 model took into account negative impacts on the ecosystem. Two options for the post-used management of plastics, polymers, and elements were considered. Photovoltaic panels had the biggest negative effect over its life cycle due to landfill (7.07 × 10^5^ Pt). Recycling photovoltaic panels resulted in the largest positive impact (−2.99 × 10^5^ Pt).

### 3.2. Human Health

**Figure 7:**
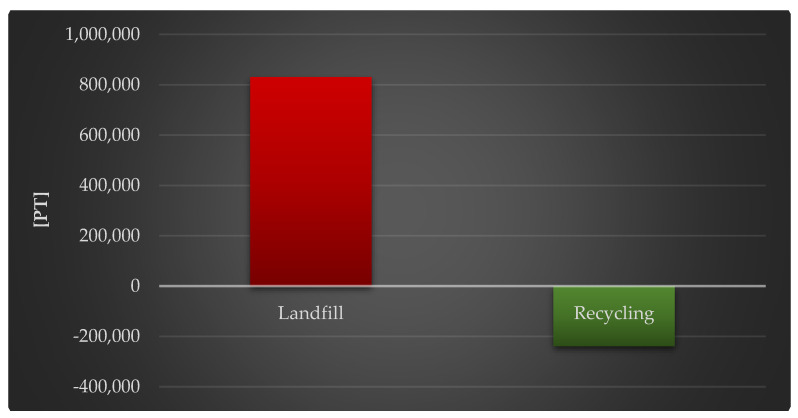
Grouping and weighting of the total consequences for the environment of the analyzed photovoltaic power plant’s life cycle, regarding their impact on human health, taking into account the method of the post-used management of materials, components, and elements [unit: Pt].

A Figure 7 shows the complete impact of a photovoltaic power plant’s life cycle, including post-used management (landfill and recycling), on human health. It is clear that adopting recycling techniques to manage the power plant’s materials, components, and working units over their full life cycle provides a variety of benefits, lowering their detrimental impact in the investigated region (by a total of 2.39 × 10^5^ Pt).

Table 3 summarizes the findings of grouping and weighting the environmental consequences of the photovoltaic power plant’s life cycle. Negative impacts on human health were considered. The operations involving the use of water in turbines had the largest negative impact (8.94 × 10^5^ Pt throughout the life cycle, with storage management). These methods were also differentiated by the highest level of positive effects for recycling as a type of post-use management (−1.90 × 10^5^ points). The findings indicate that inadequate access to water is presently one of the world’s most critical health, economic, and environmental issues. Societies exploit existing water resources in an excessive and unsustainable manner. They also drain enormous amounts of land (for example, as part of mine building). Currently, water extraction exceeds the growth in resources. Another issue is that its quality is constantly deteriorating due to pollution from sources such as industry, transportation, low emissions, agriculture, detergents, or badly guarded or illegal landfills. In underdeveloped nations, a lack of access to clean water is the root cause of many illnesses. As a result, water consumption must be carefully monitored in all industrial operations, including those involved in the life cycles of photovoltaic power plants.

**Figure 8:**
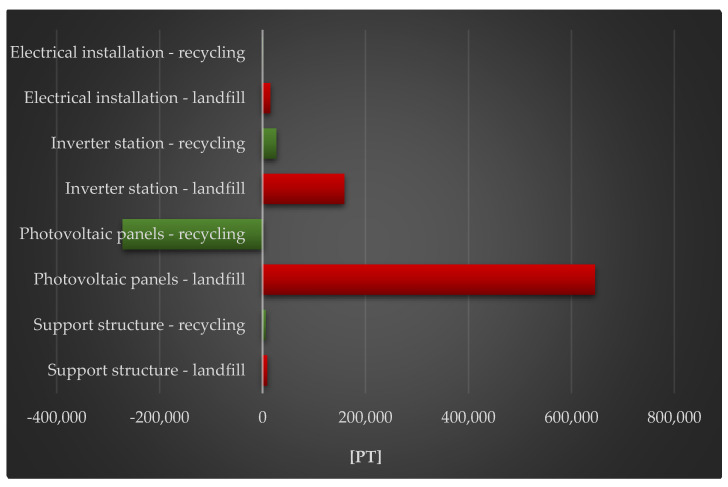
Grouping and weighting the total consequences for the environment of individual units of the analyzed photovoltaic power plant, regarding their impact on human health, taking into account the method of the post-used management of materials, components, and elements [unit: Pt].

Figure 8 shows the complete impact of a photovoltaic power plant’s life cycle, including post-used management (landfill and recycling), on human health. The largest degree of negative consequences in the investigated region is shown in the photovoltaic panel life cycle, with post-used management in the form of a landfill. Large quantities of energy are required to make photovoltaic modules (for example, photovoltaic cells produced using the Czochralski process). It is often derived from nonrenewable sources, and its usage is related to toxic substance emissions, which have an impact not only on human health, but also on environmental quality. The techniques of obtaining fossil fuels (whose supplies are depleting year after year) also have a severe influence on the environment, causing irreversible changes in land structure, such as forest clearing, drying out neighboring areas, and so on. Recycling photovoltaic modules might help to reduce these negative effects during their full life cycle. However, there is currently a scarcity of sustainable recycling processes that are beneficial not only in ecological, but also in economic and social, dimensions.

Table 4 shows the results of grouping and weighting the environmental implications of the photovoltaic power plant’s separate unit life cycles. Negative impacts on human health were considered. In this example, the highest level of negative effect was defined by processes involving the use of water in turbines (6.09 × 10^5^ Pt for the life cycle of photovoltaic panels with storage). These procedures were also differentiated by the highest degree of positive effect on the usage of recycling as a type of post-used management (−1.80 × 10^5^ Pt). The expanding global population has led to an increase in demand for water, not just for food preparation, cleanliness, and agricultural usage, but also for industrial production and services. However, these demands are not always addressed. Many people suffer and become ill as a result of a lack of access to clean water, particularly in poor nations. The issue includes a lack of access to water, as well as access to low-quality water that may contain pesticides or hazardous germs. As a result, sustainable water management is becoming increasingly crucial, even throughout the life cycles of technological installations such as photovoltaic power plants. Particular emphasis should be devoted to the use of materials and processes that consume the least amount of water feasible, as well as work on the development of new techniques and technologies that allow for the conservation of water resources.

### 3.3. Ecosystems

**Figure 9:**
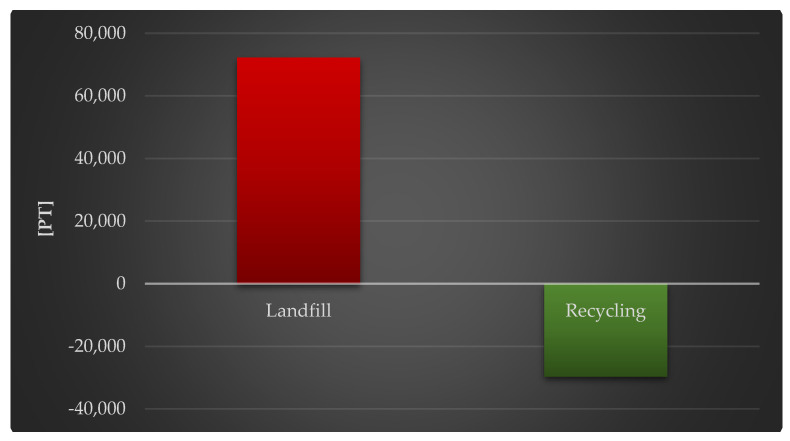
Grouping and weighting the total consequences for the environment of the life cycle of the analyzed photovoltaic power plant, regarding their impact on the ecosystem, taking into account the method of post-used management of materials, components, and elements [unit: Pt].

Figure 9 shows the whole impact of a photovoltaic power plant’s life cycle, including post-use management (landfill and recycling), on the environment. In terms of the influence on the natural environment as well as human health quality, appropriate post-use management of materials, materials, components, and functioning units of photovoltaic power plants plays a critical role. Their recycling provides for a large decrease in the negative influence on ecosystems (by a total of 2.97 × 10^4^ Pt).

Table 5 summarizes the findings of grouping and weighting the environmental implications of the photovoltaic power plant’s life cycle. Negative impacts on the ecology were considered. The operations involving the use of water in turbines had the biggest negative impact (8.91 × 10^4^ Pt during the life cycle, with storage management). These methods were also characterized by having the most beneficial impact when recycling was used as a type of post-use management (−1.96 × 10^4^ Pt). The findings show the seriousness of the issue of declining drinking water resources, as well as the major influence of human activities on its quality. The steady growth in population, urbanization, and industrial expansion causes a variety of climatic changes, one of which is the depletion of water supplies. A rising proportion of the world’s population is feeling the impact of its scarcity year after year. Water scarcity is undeniably a massive issue that needs coordinated, ethical solutions. They must be implemented not just at the governmental level, but also in firms that manufacture, run, and manage technological infrastructure, such as photovoltaic power plants. Everyone should use this incredibly important raw material properly.

**Figure 10:**
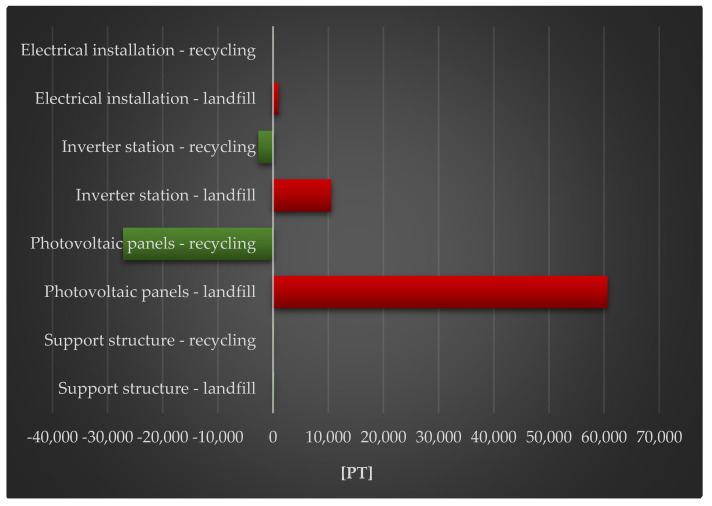
Grouping and weighting the total consequences for the environment of individual units of the analyzed photovoltaic power plant, regarding their impact on the ecosystem, taking into account the method of post-used management of materials, components, and elements [unit: Pt].

Figure 10 shows the complete impact of a photovoltaic power plant’s life cycle, including post-used management (landfill and recycling) on the environment. The most negative consequences are obvious in the investigated area for the photovoltaic panel life cycle, with post-used management in the form of landfill storage. The manufacturing and post-used management of materials of photovoltaic panel elements are extremely energy- and material-intensive, resulting in high levels of chemical substance emissions with a negative impact on the environment and a decrease in their quality.

Table 6 shows the results of grouping and weighting the environmental implications of the photovoltaic power plant’s separate unit life cycles. Negative impacts on the ecology were considered. In this example, the highest level of negative effects was defined by processes involving the use of water in turbines (6.01 × 10^4^ Pt over the life cycle of photovoltaic panels with landfill). These methods were also separated by the maximum amount of beneficial effects when recycling was used as a type of post-use management (−2.65 × 10^4^ points). Agriculture, energy generation, and manufacturing are the most water-intensive sectors. In the case of industry, the quest for savings should begin with determining which operations and how much water are consumed. One way to balance the life cycles of technical facilities (including photovoltaic power plants) in this area is to redesign technological processes so that water can be used (to the greatest extent possible) in a closed circuit (e.g., through sewage treatment or recirculation of so-called “grey water”).

### 3.4. Raw Material Resources

**Figure 11:**
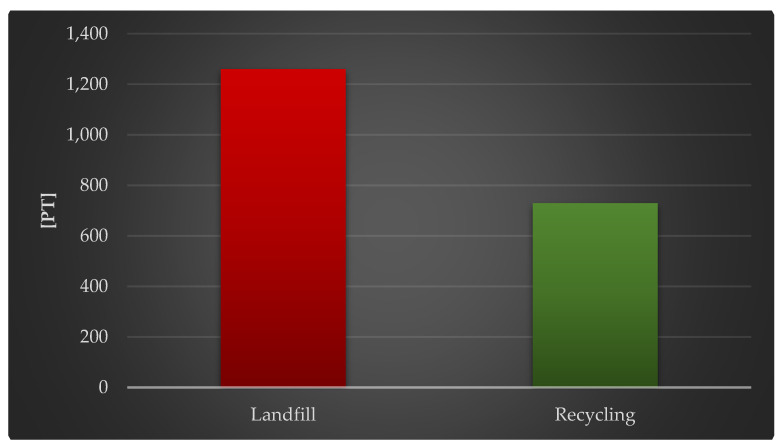
Grouping and weighting the total consequences for the environment of the life cycle of the analyzed photovoltaic power plant, regarding their impact on the depletion of raw materials, taking into account the method of the post-used management of materials, components, and elements [unit: Pt].

Figure 11 shows the complete impact of a photovoltaic power plant’s life cycle, including post-use management (landfill, recycling) and raw material depletion. The appropriate post-use management of materials, components, and functioning units of photovoltaic power plants is crucial, just as it is in regard to the environment and human health. Recycling techniques greatly minimize the detrimental impact of raw-material-related activities (1.26 × 10^3^ Pt against 7.29 × 10^2^ Pt).

Table 7 summarizes the findings of grouping and weighting the environmental implications of the photovoltaic power plant’s life cycle. Negative implications, such as the depletion of raw material supplies, were considered. The highest degree of negative effect was found in operations involving crude oil extraction and subsequent usage (3.93 × 10^1^ Pt throughout the life cycle including landfill). These methods were also separated by the maximum amount of positive effects when recycling was used as a type of post-use management (−1.27 × 10^1^ Pt). The rising consumption of crude oil not only depletes this nonrenewable energy source, but it also generates a slew of environmental concerns and a decline in environmental quality. Crude oil is a vital raw commodity in the world economy. It is employed not only in the energy industry, but also as a foundation for the manufacturing of gasoline and polymer products. Extraction, processing, refining, storage, transportation, and distribution are all aspects of the oil and product supply sector. It is also important to maintain the infrastructure supporting these operations, which includes mines, refineries, pipelines, warehouses, gasoline stations, and tankers. It is, therefore, a highly developed business that contributes to the release of pollutants into the environment (particularly in oil extraction regions), such as excessive carbon dioxide emissions, oil spills, and groundwater contamination.

**Figure 12:**
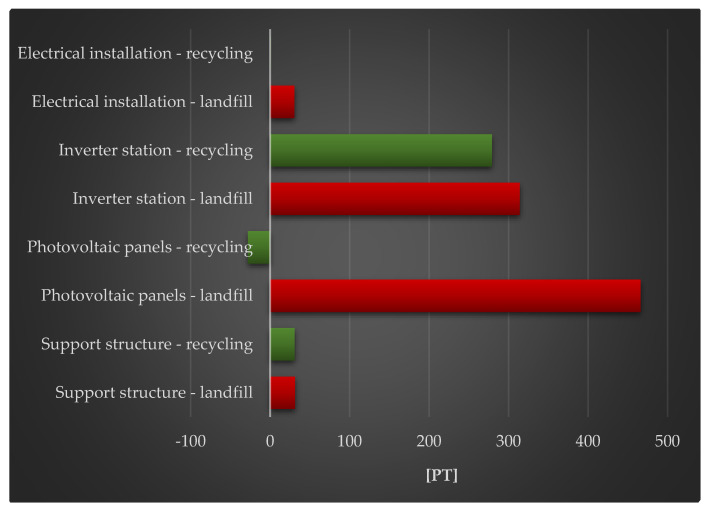
Grouping and weighting the total consequences for the environment of individual units of the analyzed photovoltaic power plant, the impact on the depletion of raw material resources, taking into account the method of post-used management of materials, components, and elements [unit: Pt].

Figure 12 shows the complete impact of a photovoltaic power plant’s life cycle, including post-used management (landfill, recycling) and raw material depletion. The largest degree of negative consequences can be shown in the investigated area for the photovoltaic panels and inverter station life cycle, with post-used management in the form of landfill. The production of cables and other electrical components (made of copper, rare earth metals, etc.) require the use of vast amounts of energy and matter, which has a substantial impact on raw material depletion. Their life cycle necessitates the implementation of improvements that are consistent with the principles of sustainable development.

Table 8 shows the results of grouping and weighting the environmental consequences of the photovoltaic power plant’s various components over time. This study includes negative implications, such as the depletion of basic materials. In this scenario, the largest amount of negative effects was associated with activities that used crude oil (2.47 × 10^2^ Pt over the life cycle of photovoltaic panels with landfill management). These methods were also characterized by the maximum degree of beneficial impacts when recycling was used for post-used management (−4.39 × 10^1^ Pt). In the case of crude oil extraction, open-pit mining poses the biggest environmental risk, as it has more negative impacts than previous extraction methods. Oil sands may now be exploited in the form of open-pit mining. Techniques for extracting these raw minerals create almost irreparable harm. Obtaining oil from an open-pit mine often entails first clearing the forest, then removing the dirt and peat that covers the oil sands, and then extracting the oil sand. Furthermore, water utilized in the oil extraction process is a byproduct that is frequently found near the mine (in the form of a reservoir that endangers the environment and human health). Post-production sewage from open-pit oil sand mines contains a variety of carcinogenic chemicals that can contaminate groundwater and harm human health.

### 3.5. Construction Materials

The final stage of this study assessed the impact of the four construction materials of the photovoltaic power plant on the environment, which had the greatest negative impact per kg: copper, solar silicon, aluminum, and steel.

**Figure 13:**
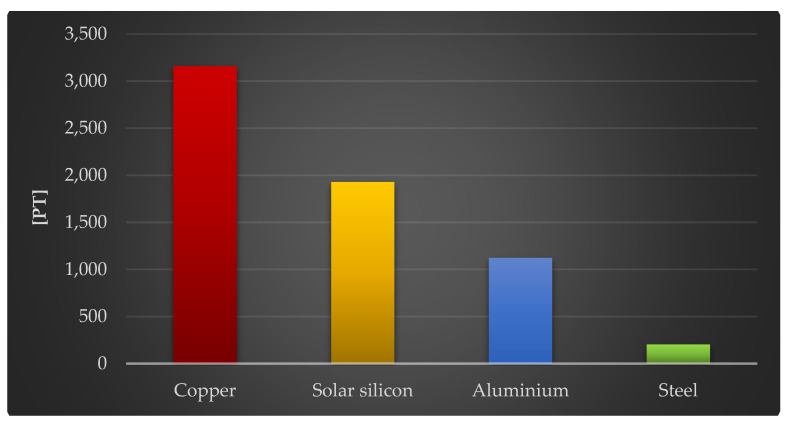
Results of grouping and weighting the total environmental consequences of the life cycles of 1000 kg of selected plastics and materials included in the components of a photovoltaic power plant [unit: Pt].

Figure 13 depicts the results of grouping and weighing the overall environmental impact of 1000 kg of the chosen polymers and materials used in photovoltaic power plant components. Additional investigations were conducted to determine which materials had the greatest harmful environmental impact. Copper, which is primarily used in electrical installations (3157 Pt/1000 kg), solar glass, which is primarily used in PV panels (1927 Pt/1000 kg), aluminum, which is primarily used in the support structure (1122 Pt/1000 kg), and steel, which is also primarily used in the support structure (204 Pt/1000 kg), had the greatest negative environmental impacts.

**Figure 14:**
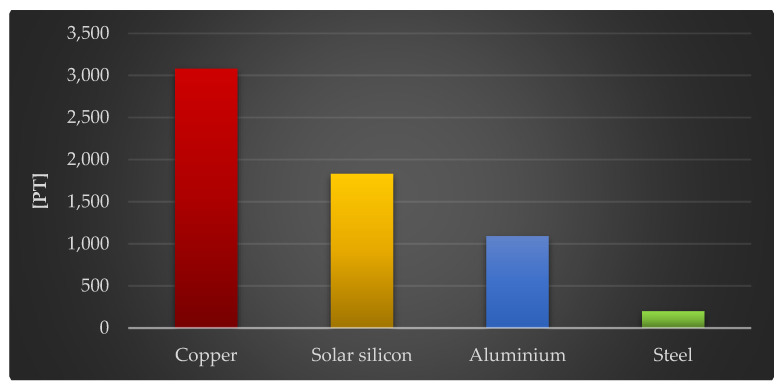
Results of grouping and weighting environmental consequences for human health in the life cycles of 1000 kg of selected plastics and materials included in the components of a photovoltaic power plant [unit: Pt].

Figure 14 depicts the findings of similar investigations, but solely in regard to the level of influence on human health. An equivalent link can be seen when considering the total environmental effect. Copper (3080 Pt/1000 kg), solar glass (1830 Pt/1000 kg), and aluminum (1090 Pt/1000 kg) have the most detrimental influence in this respect. Of the three categories of influence studied (human health, the ecosystem, and raw material resources), the analyzed materials had the most detrimental impact on human health. The extraction and processing of metal ore deposits, notably copper, results in the emission of large amounts of chemical compounds into the environment. Some of the elements are introduced into the aquatic environment via discharged mine waters and sewage from smelters and enrichment plants, while others enter the atmosphere via dust emitted by smelters, and yet another portion, contained in smelter and flotation waste, can penetrate the immediate environment after being deposited in a landfill. Copper recycling is a very effective and energy-efficient method of reintroducing this commodity into the economic cycle. It takes far less energy than primary production. The global demand for refined copper is steadily growing. Ensuring the availability of sufficient amounts of this element will necessitate, among other things, increased recovery and recycling rates.

**Figure 15:**
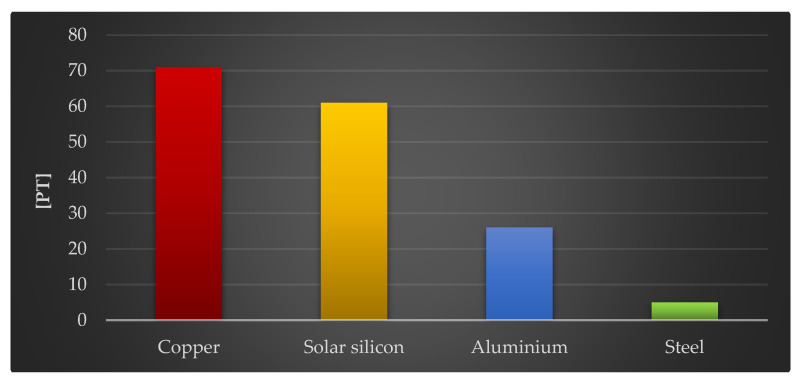
Results of grouping and weighting environmental consequences for ecosystems in the life cycles of 1000 kg of selected plastics and materials included in the components of a photovoltaic power plant [unit: Pt].

Figure 15 depicts how the tested materials affect the quality of ecosystems. Copper (71 Pt/1000 kg), solar glass (61 Pt/1000 kg), and aluminum (26 Pt/1000 kg) have the greatest negative influence on the environment, just as they do on human health. However, their negative impact on ecosystem quality is far lesser than that on human health.

**Figure 16:**
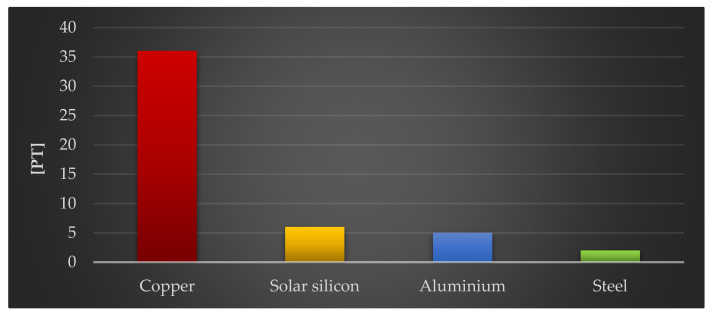
Results of grouping and weighting the environmental consequences for the depletion of raw materials in the life cycles of 1000 kg of selected plastics and materials included in the components of a photovoltaic power plant [unit: Pt].

The final figure (Figure 16) depicts the influence of the examined materials on the depletion of raw materials. In this example, the highest amount of unfavorable effects is clearly associated with the life cycle of solar glass (36 Pt/1000 kg), which is utilized in photovoltaic panels. As previously stated, its production techniques are among the most energy- and material-intensive. Among the effect areas studied (human health, the ecosystem, and raw material resources), the analyzed materials had the largest negative influence on the area of raw material depletion.

## 4. Discussion and Conclusions

The aim of this study was achieved by conducting an assessment of the environmental life cycle of materials, components, and elements of a mono-Si photovoltaic power plant towards their sustainable development.

The basic purpose of sustainable development is to meet humanity’s needs while considering future generations’ demands. The socioeconomic growth of highly developed countries involves rapid social and economic development while simultaneously improving the population’s quality of life and the environment. These assumptions are addressed by the concepts of life cycle thinking (LCT) and life cycle management (LCM). Their use is intended to reduce the negative environmental impact of photovoltaic power plant manufacturing, operation, and post-used management operations. Environmental Life Cycle Assessment (LCA) is the primary tool for conducting analytical work in this domain, allowing for the quantitative measurement of the magnitude of potential consequences at each stage [42].

This study uses the ReCiPe 2016 model to undertake a complete assessment of the environmental effects of a photovoltaic power plant’s life cycle, taking into consideration three important areas of impact: human health, ecosystem quality, and raw material depletion. An analysis of the results for different groups of power plant elements, such as the support structure, PV panels, inverter station, and electrical installation, reveals significant differences in the level of negative and positive impacts depending on the materials’ post-used management scenario.

Waste storage had the largest detrimental impact on human health (8.30 × 10^5^ Pt); however, recycling helped to significantly reduce this impact (−2.39 × 10^5^ Pt). A similar association was seen with photovoltaic panels, where storage had a negative impact of 7.07 × 10^5^ Pt, but recycling had considerable environmental advantages (−2.99 × 10^5^ Pt). In turn, the support structure had the least negative influence on the environment, owing to its material composition (mostly steel and aluminum), which is more easily reused.

The most detrimental impact on ecosystems was induced by procedures involving the use of water in turbines (8.94 × 10^5^ Pt—landfill). At the same time, recycling methods had the biggest beneficial impact in this regard (−1.90 × 10^5^ Pt). These findings support the rising relevance of sustainable water resource management, particularly in industrial and energy sectors. Excessive water extraction and pollution are severe threats to the environment and public health, necessitating the deployment of technologies that limit water use and promote closed circulation.

The production and consumption of crude oil has been identified as a major contributor to raw material depletion. The highest level of crude oil consumption, and therefore the maximum level of harmful impact, was recorded in the life cycle of photovoltaic panels at 2.47 × 10^2^ Pt (management in the form of landfill); however, recycling would reduce this impact to −4.39 × 10^1^ Pt because of the reuse of some materials. These findings clearly demonstrate the need to minimize crude oil consumption by utilizing renewable energy sources and improving the efficiency of material recycling.

During the life cycle of a photovoltaic power plant (especially PV panels), a particularly large number of harmful substances are emitted into the environment, which have a negative impact on human health (mainly as a consequence of the high demand for energy and material at the production stage and the insufficient level of recycling at the post-used management stage).

This study demonstrates that using recycling as a type of post-used material management greatly minimizes a photovoltaic power plant’s negative environmental effect in all regions studied. Furthermore, the findings indicate the critical necessity to establish a plan for sustainable water and raw material management (especially copper) throughout the life cycle of technological facilities in order to reduce environmental impact. It is necessary to reduce energy and material consumption throughout the entire life cycle of photovoltaic power plants (especially PV panels). It is recommended that further technologies be developed to decrease emissions and enhance recycling operations, hence increasing the energy and environmental efficiency of photovoltaic systems.

The exploitation stage in the life cycle of photovoltaic power plants best fits with the main assumptions of sustainable development. In the manufacturing stage, there is still work to be done to reduce the consumption of energy, matter, and water. It is necessary to introduce new manufacturing techniques and materials whose life cycles will have fewer negative effects on the environment. Among the available post-used management scenarios, the most sustainable is the use of recycling processes. In the life cycle of the materials, components, and elements of photovoltaic power plants, activities in the field of the economy in a (possibly) closed loop must be introduced.

## Figures and Tables

**Table 1:** Grouping and weighting the consequences for the environment of the life cycle of the analyzed photovoltaic power plant, regarding the effects of their emissions on human health, the ecosystem, and raw material resources, taking into account the areas of impact (ReCiPe 2016 model) and the method of the post-used management of materials, components, and elements [unit: Pt].

No	Element of a Technical Object	Photovoltaic Power Plant	
	Waste Scenario	Landfill	Recycling
	Impact Category		
1	Human health	8.30 × 10^5^	−2.39 × 10^5^
2	Ecosystems	7.22 × 10^4^	−2.97 × 10^4^
3	Raw material resources	1.26 × 10^3^	7.29 × 10^2^
**Total**		**9.03 × 10^5^**	**−2.68 × 10^5^**

**Table 2:** Grouping and weighting the consequences for the environment of the life cycle of individual units of the analyzed photovoltaic power plant (supporting structure, photovoltaic panels, inverter station and electrical installation), regarding their impact on human health, the ecosystem, and raw material resources. The ReCiPe 2016 model took into account the method of post-used management of materials, components, and elements [unit: Pt].

No	Element of a Technical Object	Support Structure		Photovoltaic Panels		Inverter Station		Electrical Installation	
	Waste Scenario	Landfill	Recycling	Landfill	Recycling	Landfill	Recycling	Landfill	Recycling
	Impact Category								
1	Human health	8.97 × 10^3^	6.19 × 10^3^	6.46 × 10^5^	−2.72 × 10^5^	1.59 × 10^5^	2.69 × 10^4^	1.52 × 10^4^	9.56 × 10^1^
2	Ecosystems	1.93 × 10^2^	1.14 × 10^2^	6.06 × 10^4^	−2.72 × 10^4^	1.05 × 10^4^	−2.66 × 10^3^	9.49 × 10^2^	5.97 × 10^0^
3	Raw material resources	3.13 × 10^1^	3.08 × 10^1^	4.16 × 10^2^	−2.38 × 10^1^	7.87 × 10^2^	7.22 × 10^2^	3.05 × 10^1^	1.92 × 10^1^
	**Total**	**9.20** × **10^3^**	**6.34** × **10^3^**	**7.07** × **10^5^**	**−2.99** × **10^5^**	**1.71** × **10^5^**	**2.50** × **10^4^**	**1.62** × **10^4^**	**1.02** × **10^2^**

**Table 3:** Grouping and weighting the consequences for the environment of the life cycle of the analyzed photovoltaic power plant, regarding their impact on human health, taking into account the method of post-used management of materials, components, and elements [unit: Pt].

No	Element of a Technical Object		Photovoltaic Power Plant	
	Waste Scenario		Landfill	Recycling
	Substance	Emission Area		
1	Ammonia	Air	2.70 × 10^2^	1.63 × 10^2^
2	Antimony	Air	3.52 × 10^0^	1.36 × 10^0^
3	Antimony	Water	5.26 × 10^0^	4.99 × 10^0^
4	Arsenic	Air	1.24 × 10^3^	7.54 × 10^2^
5	Arsenic	Water	1.05 × 10^4^	8.35 × 10^3^
6	Barium	Water	2.38 × 10^2^	2.17 × 10^2^
7	Benzo(a)pyrene	Air	x	8.03 × 10^−1^
8	Beryllium	Air	x	5.18 × 10^0^
9	Cadmium	Air	1.30 × 10^2^	7.22 × 10^1^
10	Cadmium	Water	2.80 × 10^1^	2.57 × 10^1^
11	Carbon-14	Air	x	2.28 × 10^0^
12	Carbon dioxide, fossil	Air	1.91 × 10^4^	9.92 × 10^3^
13	Carbon dioxide, land transformation	Air	5.27 × 10^1^	5.27 × 10^1^
14	Carbon disulfide	Air	8.23 × 10^1^	2.76 × 10^1^
15	Chromium VI	Air	3.66 × 10^1^	5.93 × 10^1^
16	Chromium VI	Water	1.52 × 10^4^	5.51 × 10^3^
17	Chromium VI	Soil	1.42 × 10^2^	1.31 × 10^2^
18	Copper	Water	x	5.50 × 10^0^
19	Dinitrogen monoxide	Air	3.02 × 10^2^	1.92 × 10^2^
20	Dioxin, 2,3,7,8 Tetrachlorodibenzo-p-	Air	2.09 × 10^0^	2.85 × 10^0^
21	Ethane, hexafluoro-, HFC-116	Air	3.12 × 10^2^	−1.46 × 10^2^
22	Formaldehyde	Air	x	2.85 × 10^0^
23	Hydrocarbons, chlorinated	Air	1.74 × 10^0^	1.09 × 10^−2^
24	Lead	Air	1.32 × 10^3^	1.04 × 10^3^
25	Lead	Water	9.08 × 10^2^	8.35 × 10^2^
26	Mercury	Air	x	8.82 × 10^0^
27	Mercury	Water	2.13 × 10^2^	1.81 × 10^2^
28	Methane, biogenic	Air	1.76 × 10^3^	1.30 × 10^3^
29	Methane, chlorodifluoro-, HCFC-22	Air	1.57 × 10^0^	1.57 × 10^0^
30	Methane, fossil	Air	1.68 × 10^3^	8.68 × 10^2^
31	Methane, tetrafluoro-, CFC-14	Air	1.66 × 10^3^	−7.79 × 10^2^
32	Nickel	Air	4.73 × 10^1^	1.48 × 10^0^
33	Nickel	Water	1.87 × 10^1^	4.89 × 10^1^
34	Nitrogen oxides	Air	4.47 × 10^3^	2.32 × 10^3^
35	Particulates, <2.5 mm	Air	9.73 × 10^3^	3.92 × 10^3^
36	Radon-222	Air	2.48 × 10^1^	2.02 × 10^1^
37	Silver	Water	2.60 × 10^1^	2.60 × 10^1^
38	Sulfur dioxide	Air	2.16 × 10^4^	1.01 × 10^4^
39	Sulfur hexafluoride	Air	2.61 × 10^2^	2.55 × 10^2^
40	Sulfur oxides	Air	x	6.89 × 10^0^
41	Sulfur trioxide	Air	2.63 × 10^2^	2.63 × 10^2^
42	Thallium	Water	7.76 × 10^1^	1.07 × 10^2^
43	Vanadium	Water	1.46 × 10^2^	6.07 × 10^1^
44	Water (total)	Water	−1.90 × 10^5^	−1.90 × 10^5^
45	Water, cooling, unspecified natural origin (total)	Raw materials	2.09 × 10^3^	1.48 × 10^3^
46	Water, lake (total)	Raw materials	x	1.76 × 10^1^
47	Water, river (total)	Raw materials	2.42 × 10^2^	2.57 × 10^2^
48	Water, turbine use, unspecified natural origin (total)	Raw materials	8.94 × 10^5^	−1.25 × 10^5^
49	Water, unspecified natural origin (total)	Raw materials	5.86 × 10^2^	5.74 × 10^2^
50	Water, well (total)	Raw materials	4.89 × 10^1^	6.73 × 10^1^
51	Zinc	Air	4.02 × 10^2^	3.13 × 10^2^
52	Zinc	Water	2.87 × 10^4^	2.55 × 10^4^
53	Zinc	Soil	9.51 × 10^1^	9.67 × 10^1^
54	Remaining substances	x	6.61 × 10^2^	5.47 × 10^3^
**Total**			**8.30 × 10^5^**	**−2.39 × 10^5^**

**Table 4:** Grouping and weighting the consequences for the environment of the life cycle of individual units of the analyzed photovoltaic power plant, in the area of impact on human health, taking into account the method of post-used management of materials, components, and elements [unit: Pt].

No	Element of a Technical Object		Support Structure		Photovoltaic Panels		Inverter Station		Electrical Installation	
	Waste Scenario		Landfill	Recycling	Landfill	Recycling	Landfill	Recycling	Landfill	Recycling
	Impact Category	Emission Area								
1	Ammonia	Air	1.16 × 10^1^	1.13 × 10^1^	1.09 × 10^2^	5.99 × 10^1^	9.85 × 10^1^	9.12 × 10^1^	5.12 × 10^1^	3.22 × 10^−1^
2	Antimony	Air	1.34 × 10^0^	1.34 × 10^0^	-	-	-	3.78 × 10^−3^	2.18 × 10^0^	1.37 × 10^−2^
3	Antimony	Water	5.26 × 10^0^	4.88 × 10^0^	-	-	-	1.11 × 10^−1^	-	-
4	Arsenic	Air	3.83 × 10^0^	3.78 × 10^0^	1.08 × 10^2^	9.41 × 10^1^	6.51 × 10^2^	6.53 × 10^2^	4.79 × 10^2^	3.01 × 10^0^
5	Arsenic	Water	8.37 × 10^1^	7.18 × 10^1^	1.29 × 10^3^	1.30 × 10^2^	7.98 × 10^3^	8.14 × 10^3^	1.14 × 10^3^	7.17 × 10^0^
6	Barium	Water	6.66 × 10^0^	1.14 × 10^0^	1.21 × 10^2^	1.15 × 10^2^	1.02 × 10^2^	1.01 × 10^2^	8.13 × 10^0^	5.11 × 10^−2^
7	Benzo(a)pyrene	Air	-	8.03 × 10^−1^	-	-	-	-	-	-
8	Beryllium	Air	-	-	-	-	-	5.18 × 10^0^	-	-
9	Cadmium	Air	3.22 ×10^0^	-	-	-	7.18 × 10^1^	7.18 × 10^1^	5.53 × 10^1^	3.48 × 10^−1^
10	Cadmium	Water	-	-	-	-	2.64 × 10^1^	2.57 × 10^1^	1.59 × 10^0^	1.00 × 10^−2^
11	Carbon-14	Air	-	-	-	-	-	2.28 × 10^0^	-	-
12	Carbon dio-ide, fossil	Air	1.32 × 10^3^	1.31 × 10^3^	8.98 × 10^3^	1.69 × 10^3^	8.52 × 10^3^	6.92 × 10^3^	2.63 × 10^2^	1.65 × 10^0^
13	Carbon dio-ide, land transformation	Air	9.75 × 10^−1^	9.75 × 10^−1^	-	-	5.17 × 10^1^	5.17 × 10^1^	-	-
14	Carbon disulfide	Air	-	-	-	-	2.72 × 10^1^	2.73 × 10^1^	5.51 × 10^1^	3.47 × 10^−1^
15	Chromium VI	Air	-	-	-	2.92 × 10^1^	3.07 × 10^1^	3.01 × 10^1^	5.87 × 10^0^	3.69 × 10^−2^
16	Chromium VI	Water	2.68 × 10^3^	2.65 × 10^3^	6.27 × 10^3^	−2.02 × 10^3^	6.12 × 10^3^	4.88 × 10^3^	9.10 × 10^1^	5.72 × 10^−1^
17	Chromium VI	Soil	-	-	-	-	1.42 × 10^2^	1.31 × 10^2^	-	-
18	Copper	Water	-	-	-	-	-	5.50 × 10^0^	-	-
19	Dinitrogen mono-ide	Air	6.01 × 10^0^	4.97 × 10^0^	8.81 × 10^1^	-	2.00 × 10^2^	1.87 × 10^2^	8.06 × 10^0^	5.07 × 10^−2^
20	Dio-in, 2,3,7,8 Tetrachlorodibenzo-p-	Air	-	-	-	-	-	2.84 × 10^0^	2.09 × 10^0^	1.31 × 10^−2^
21	Ethane, he-afluoro-, HFC-116	Air	-	-	2.71 × 10^2^	−1.27 × 10^2^	4.10 × 10^1^	−1.88 × 10^1^	-	-
22	Formaldehyde	Air	-	-	-	-	-	2.85 × 10^0^	-	-
23	Hydrocarbons, chlorinated	Air	-	-	-	-	-	-	1.74 × 10^0^	1.09 × 10^−2^
24	Lead	Air	7.30 × 10^0^	7.26 × 10^0^	1.18 × 10^2^	1.06 × 10^2^	9.25 × 10^2^	9.27 × 10^2^	2.72 × 10^2^	1.71 × 10^0^
25	Lead	Water	3.99 × 10^1^	×	2.54 × 10^2^	2.50 × 10^2^	5.96 × 10^2^	5.85 × 10^2^	1.85 × 10^1^	1.16 × 10^−1^
26	Mercury	Air	-	-	-	-	-	8.82 × 10^0^	-	-
27	Mercury	Water	1.89 × 10^1^	1.95 × 10^0^	8.16 × 10^1^	8.02 × 10^1^	1.04 × 10^2^	9.92 × 10^1^	8.23 × 10^0^	5.18 × 10^−2^
28	Methane, biogenic	Air	5.67 × 10^2^	7.33 × 10^−1^	9.32 × 10^2^	9.30 × 10^2^	1.92 × 10^2^	3.69 × 10^2^	6.47 × 10^1^	4.07 × 10^−1^
29	Methane, chlorodifluoro-, HCFC-22	Air	1.57 × 10^0^	1.57 × 10^0^	-	-	-	-	-	-
30	Methane, fossil	Air	2.00 × 10^2^	1.72 × 10^2^	6.64 × 10^2^	-	7.85 × 10^2^	6.96 × 10^2^	2.72 × 10^1^	1.71 × 10^−1^
31	Methane, tetrafluoro-, CFC-14	Air	-	-	1.44 × 10^3^	−6.79 × 10^2^	2.19 × 10^2^	−9.96 × 10^1^	-	-
32	Nickel	Air	1.17 × 10^0^	1.17 × 10^0^	-	-	2.27 × 10^−2^	2.29 × 10^−2^	4.61 × 10^1^	2.90 × 10^−1^
33	Nickel	Water	1.12 × 10^1^	3.90 × 10^0^	-	4.36 × 10^1^	1.39 × 10^0^	1.37 × 10^0^	6.06 × 10^0^	3.81 × 10^−2^
34	Nitrogen o-ides	Air	2.51 × 10^2^	2.47 × 10^2^	1.81 × 10^3^	1.01 × 10^2^	2.22 × 10^3^	1.97 × 10^3^	1.86 × 10^2^	1.17 × 10^0^
35	Particulates, <2.5 mm	Air	1.01 × 10^3^	1.00 × 10^3^	3.56 × 10^3^	−2.74 × 10^2^	3.76 × 10^3^	3.19 × 10^3^	1.40 × 10^3^	8.81 × 10^0^
36	Radon-222	Air	-	-	-	-	2.48 × 10^1^	2.02 × 10^1^	-	-
37	Silver	Water	-	-	-	-	2.60 × 10^1^	2.60 × 10^1^	-	-
38	Sulfur dio-ide	Air	4.92 × 10^2^	4.85 × 10^2^	7.77 × 10^3^	−7.96 × 10^2^	1.17 × 10^4^	1.04 × 10^4^	1.62 × 10^3^	1.02 × 10^1^
39	Sulfur he-afluoride	Air	9.92 × 10^−1^	9.79 × 10^−1^	-	-	2.60 × 10^2^	2.54 × 10^2^	-	-
40	Sulfur o-ides	Air	-	-	-	-	-	6.89 × 10^0^	-	-
41	Sulfur trio-ide	Air	-	-	-	-	2.63 × 10^2^	2.63 × 10^2^	-	-
42	Thallium	Water	-	7.15 × 10^−1^	-	3.27 × 10^1^	7.37 × 10^1^	7.37 × 10^1^	3.92 - 10^0^	2.47 - 10^−2^
43	Vanadium	Water	4.59 × 10^1^	4.55 × 10^1^	7.08 × 10^1^	−2.93 × 10^1^	2.95 × 10^1^	4.45 × 10^1^	-	-
44	Water (total)	Water	−2.86 × 10^3^	−2.87 × 10^3^	−6.22 × 10^3^	−6.73 × 10^3^	−1.80 × 10^5^	−1.80 × 10^5^	−7.18 × 10^2^	−4.51 × 10^0^
45	Water, cooling, unspecified natural origin (total)	Raw materials	2.82 × 10^1^	2.88 × 10^1^	4.95 × 10^2^	−4.83 × 10^1^	1.56 × 10^3^	1.50 × 10^3^	8.53 × 10^0^	5.36 × 10^−2^
46	Water, lake (total)	Raw materials	-	-	-	-	-	1.76 × 10^1^	-	-
47	Water, river (total)	Raw materials	9.89 × 10^−1^	9.89 × 10^−1^	-	-	2.32 × 10^2^	2.56 × 10^2^	8.59 × 10^0^	5.40 × 10^−2^
48	Water, turbine use, unspecified natural origin (total)	Raw materials	3.08 × 10^3^	2.86 × 10^3^	6.09 × 10^5^	−2.69 × 10^5^	2.73 × 10^5^	1.41 × 10^5^	9.34 × 10^3^	5.87 × 10^1^
49	Water, unspecified natural origin (total)	Raw materials	4.97 × 10^0^	4.97 × 10^0^	-	-	5.67 × 10^2^	5.69 × 10^2^	1.39 × 10^1^	8.74 × 10^−2^
50	Water, well (total)	Raw materials	1.09 × 10^0^	1.09 × 10^0^	-	-	4.78 × 10^1^	6.62 × 10^1^	-	-
51	Zinc	Air	1.59 × 10^0^	1.57 × 10^0^	6.84 × 10^1^	3.44 × 10^1^	2.72 × 10^2^	2.77 × 10^2^	6.00 × 10^1^	3.77 × 10^−1^
52	Zinc	Water	1.93 × 10^3^	1.24 × 10^2^	7.82 × 10^3^	7.71 × 10^3^	1.83 × 10^4^	1.77 × 10^4^	6.41 × 10^2^	4.03 × 10^0^
53	Zinc	Soil	-	8.77 × 10^−1^	-	-	9.51 × 10^1^	9.58 × 10^1^	-	-
54	Remaining substances	-	1.54 × 10^1^	1.15 × 10^1^	5.56 × 10^2^	7.23 × 10^1^	7.58 × 10^1^	5.39 × 10^3^	1.39 × 10^1^	8.74 × 10^−2^
	**Total**		**8.97 × 10^3^**	**6.19 × 10^3^**	**6.46 × 10^5^**	**−2.72 × 10 ^5^**	**1.59 × 10^5^**	**2.69 × 10^4^**	**1.52 × 10^4^**	**9.56 × 10^1^**

**Table 5:** Grouping and weighting the consequences for the environment of the life cycle of the analyzed photovoltaic power plant, regarding their impact on the ecosystem, taking into account the method of the post-used management of materials, components, and elements [unit: Pt].

No	Element of a Technical Object		Photovoltaic Power Plant	
	Waste Scenario		Landfill	Recycling
	Substance	Emission Area		
1	Ammonia	Air	1.06 × 10^2^	9.58 × 10^1^
2	Antimony	Air	1.78 × 10^−1^	6.87 × 10^−2^
3	Antimony	Water	6.91 × 10^−2^	6.40 × 10^−2^
4	Arsenic	Air	2.39 × 10^−1^	3.62 × 10^−3^
5	Arsenic	Water	-	-
6	Barium	Water	-	-
7	Benzene	Air	3.25 × 10^−2^	3.25 × 10^−2^
8	Benzo(a)pyrene	Air	-	-
9	BOD5 (Biological O-ygen Demand)	Water	2.31 × 10^1^	1.25 × 10^1^
10	Cadmium	Air	2.46 × 10^−1^	2.46 × 10^−3^
11	Carbon dio-ide, fossil	Air	9.33 × 10^2^	3.19 × 10^2^
12	Carbon dio-ide, land transformation	Air	2.58 × 10^0^	2.58 × 10^0^
13	Carbon disulfide	Air	-	-
14	Chromium	Air	1.92 × 10^1^	1.92 × 10^1^
15	Chromium VI	Air	-	-
16	Chromium VI	Water	1.30 × 10^−1^	1.28 × 10^−1^
17	Chromium VI	Soil	-	-
18	COD (Chemical O-ygen Demand)	Water	7.58 × 10^1^	4.38 × 10^1^
19	Copper	Air	7.60 × 10^1^	5.74 × 10^1^
20	Copper	Water	3.86 × 10^1^	3.39 × 10^1^
21	Dinitrogen mono-ide	Air	1.03 × 10^1^	9.17 × 10^0^
22	Dio-in, 2,3,7,8 Tetrachlorodibenzo-p-	Air	-	−9.15 × 10^−1^
23	Ethane, he-afluoro-, HFC-116	Air	1.52 × 10^1^	−6.20 × 10^0^
24	Lead	Air	1.94 × 10^0^	1.50 × 10^0^
25	Mercury	Water	-	4.19 × 10^−3^
26	Methane, biogenic	Air	8.58 × 10^1^	6.35 × 10^1^
27	Methane, chlorodifluoro-, HCFC-22	Air	7.59 × 10^−2^	7.59 × 10^−2^
28	Methane, fossil	Air	8.23 × 10^1^	4.25 × 10^1^
29	Methane, tetrafluoro-, CFC-14	Air	8.14 × 10^1^	−3.81 × 10^1^
30	Nickel	Air	5.87 × 10^0^	4.48 × 10^0^
31	Nickel	Water	1.48 × 10^0^	1.40 × 10^0^
32	Nitrogen o-ides	Air	2.13 × 10^2^	1.10 × 10^2^
33	NMVOC, non-methane volatile organic compounds	Air	5.05 × 10^0^	4.47 × 10^0^
34	Occupation (total)	Raw materials	6.96 × 10^1^	6.48 × 10^1^
35	Particulates, <2.5 mm	Air	-	-
36	Phosphate	Water	1.03 × 10^2^	9.30 × 10^1^
37	Phosphorus	Water	-	1.15 × 10^−2^
38	Silver	Water	1.06 × 10^0^	1.06 × 10^0^
39	Sulfur dio-ide	Air	4.08 × 10^2^	1.91 × 10^2^
40	Sulfur he-afluoride	Air	1.27 × 10^1^	1.24 × 10^1^
41	Sulfur o-ides	Air	-	-
42	Sulfur trio-ide	Air	5.00 × 10^0^	5.00 × 10^0^
43	Thallium	Water	-	-
44	Transformation, from forest (total)	Raw materials	4.41 × 10^1^	3.76 × 10^1^
45	Transformation, from shrub (total)	Raw materials	3.45 × 10^−2^	3.71 × 10^−1^
46	Transformation, to forest (total)	Raw materials	−2.87 × 10^1^	−3.24 × 10^1^
47	Transformation, to shrub (total)	Raw materials	−3.37 × 10^−2^	−3.63 × 10^−1^
48	Vanadium	Air	2.39 × 10^−2^	2.38 × 10^−2^
49	Vanadium	Water	8.11 × 10^−1^	1.59 × 10^0^
50	Water (total)	Water	−1.96 × 10^4^	−1.96 × 10^4^
51	Water, cooling, unspecified natural origin (total)	Raw materials	2.28 × 10^2^	1.64 × 10^2^
52	Water, lake (total)	Raw materials	1.18 × 10^0^	1.73 × 10^0^
53	Water, river (total)	Raw materials	3.15 × 10^1^	2.59 × 10^1^
54	Water, turbine use, unspecified natural origin (total)	Raw materials	8.91 × 10^4^	−1.15 × 10^4^
55	Water, unspecified natural origin (total)	Raw materials	5.80 × 10^1^	5.66 × 10^1^
56	Water, well (total)	Raw materials	6.14 × 10^0^	6.73 × 10^0^
57	Zinc	Air	4.57 × 10^0^	3.69 × 10^0^
58	Zinc	Water	4.42 × 10^1^	3.94 × 10^1^
59	Zinc	Soil	-	-
60	Remaining substances	-	9.58 × 10^1^	6.11 × 10^1^
**Total**			**7.22 × 10^4^**	**−2.97 × 10^4^**

**Table 6:** Grouping and weighting the consequences for the environment of the life cycle of individual units of the analyzed photovoltaic power plant, regarding their impact on the ecosystem, taking into account the method of post-used management of materials, components, and elements [unit: Pt].

No	Element of a Technical Object		Support Structure		Photovoltaic Panels		Inverter Station		Electrical Installation	
	Waste Scenario		Landfill	Recycling	Landfill	Recycling	Landfill	Recycling	Landfill	Recycling
	Impact Category	Emission Area								
1	Ammonia	Air	5.17 × 10^−1^	5.07 × 10^−1^	-	-	1.03 × 10^2^	9.53 × 10^1^	2.29 × 10^0^	1.44 × 10^−2^
2	Antimony	Air	6.42 × 10^−2^	6.42 × 10^−2^	-	-	-	3.78 × 10^−3^	1.14 × 10^−1^	7.17 × 10^−4^
3	Antimony	Water	6.91 × 10^−2^	6.40 × 10^−2^	-	-	-	-	-	-
4	Arsenic	Air	-	-	-	-	-	2.12 × 10^−3^	2.39 × 10^−1^	1.50 × 10^−3^
5	Arsenic	Water	-	-	-	-	-	-	-	-
6	Barium	Water	-	-	-	-	-	-	-	-
7	Benzene	Air	3.25 × 10^−2^	3.25 × 10^−2^	-	-	-	-	-	-
8	Benzo(a)pyrene	Air	-	-	-	-	-	-	-	-
9	BOD5 (Biological O-ygen Demand)	Water	4.32 × 10^0^	2.95 × 10^−1^	1.13 × 10^1^	5.77 × 10^0^	6.85 × 10^0^	6.42 × 10^0^	5.83 × 10^−1^	3.67 × 10^−3^
10	Cadmium	Air	-	-	-	-	-	9.17 × 10^−4^	2.46 × 10^−1^	1.55 × 10^−3^
11	Carbon dio-ide. fossil	Air	6.44 × 10^1^	6.39 × 10^1^	4.39 × 10^2^	−8.29 × 10^1^	4.17 × 10^2^	3.38 × 10^2^	1.29 × 10^1^	8.11 × 10^−2^
12	Carbon dio-ide. land transformation	Air	4.77 × 10^−2^	4.77 × 10^−2^	-	-	2.53 × 10^0^	2.53 × 10^0^	-	-
13	Carbon disulfide	Air	-	-	-	-	-	-	-	-
14	Chromium	Air	-	-	-	-	1.92 × 10^1^	1.92 × 10^1^	-	-
15	Chromium VI	Air	-	-	-	-	-	-	-	-
16	Chromium VI	Water	1.30 × 10^−1^	1.28 × 10^−1^	-	-	-	-	-	-
17	Chromium VI	Soil	-	-	-	-	-	-	-	-
18	COD (Chemical O-ygen Demand)	Water	1.75 × 10^1^	6.24 × 10^−1^	3.56 × 10^1^	2.66 × 10^1^	2.05 × 10^1^	1.66 × 10^1^	2.18 × 10^0^	1.37 × 10^−2^
19	Copper	Air	3.03 × 10^−1^	3.02 × 10^−1^	6.34 × 10^0^	2.90 × 10^0^	5.46 × 10^1^	5.41 × 10^1^	1.47 × 10^1^	9.25 × 10^−2^
20	Copper	Water	2.39 × 10^0^	8.55 × 10^−2^	1.58 × 10^1^	1.57 × 10^1^	1.88 × 10^1^	1.8 × 10^1^	1.64 × 10^0^	1.03 × 10^−2^
21	Dinitrogen mono-ide	Air	2.87 × 10^−1^	2.38 × 10^−1^	-	-	9.58 × 10^0^	8.93 × 10^0^	3.85 × 10^−1^	2.42 × 10^−3^
22	Dio-in. 2.3.7.8 Tetrachlorodibenzo-p-	Air	-	-	-	-	-	−9.15 × 10^−1^	-	-
23	Ethane. he-afluoro-. HFC-116	Air	-	-	1.32 × 10^1^	−6.20 × 10^0^	2.00 × 10^0^	-	-	-
24	Lead	Air	-	1.15 × 10^−2^	-	-	1.48 × 10^0^	1.48 × 10^0^	4.57 × 10^−1^	2.87 × 10^−3^
25	Mercury	Water	-	-	-	-	-	4.19 × 10^−3^	-	-
26	Methane. biogenic	Air	2.77 × 10^1^	3.58 × 10^−2^	4.55 × 10^1^	4.54 × 10^1^	9.40 × 10^0^	1.80 × 10^1^	3.16 × 10^0^	1.99 × 10^−2^
27	Methane. chlorodifluoro-. HCFC-22	Air	7.59 × 10^−2^	7.59 × 10^−2^	-	-	-	-	-	-
28	Methane. fossil	Air	9.83 × 10^0^	8.43 × 10^0^	3.26 - 10^1^	-	3.85 × 10^1^	3.41 × 10^1^	1.33 × 10^0^	8.36 × 10^−3^
29	Methane. tetrafluoro-. CFC-14	Air	-	-	7.07 × 10^1^	−3.32 × 10^1^	1.07 × 10^1^	−4.88 × 10^0^	-	-
30	Nickel	Air	3.44 × 10^−2^	3.42 × 10^−2^	-	-	4.46 × 10^0^	4.43 × 10^0^	1.37 × 10^0^	8.62 × 10^−3^
31	Nickel	Water	9.00 × 10^−2^	3.12 × 10^−2^	-	-	1.39 × 10^0^	1.37 × 10^0^	-	-
32	Nitrogen o-ides	Air	1.19 × 10^1^	1.17 × 10^1^	8.60 × 10^1^	4.82 × 10^0^	1.06 × 10^2^	9.34 × 10^1^	8.83 × 10^0^	5.55 × 10^−2^
33	NMVOC. non-methane volatile organic compounds	Air	1.75 × 10^0^	1.74 × 10^0^	-	-	3.02 × 10^0^	2.73 × 10^0^	2.80 × 10^−1^	1.76 × 10^−3^
34	Occupation (total)	Raw materials	3.83 × 10^0^	3.71 × 10^0^	8.61 × 10^0^	1.04 × 10^1^	4.99 × 10^1^	5.06 × 10^1^	7.32 × 10^0^	4.60 × 10^−2^
35	Particulates. <2.5 mm	Air	-	-	-	-	-	-	-	-
36	Phosphate	Water	7.93 × 10^0^	7.82 × 10^0^	3.08 × 10^1^	2.42 × 10^1^	6.00 × 10^1^	6.10 × 10^1^	3.93 × 10^0^	2.47 × 10^−2^
37	Phosphorus	Water	-	1.15 × 10^−2^	-	-	-	-	-	-
38	Silver	Water	-	-	-	-	1.06 × 10^0^	1.06 × 10^0^	-	-
39	Sulfur dio-ide	Air	9.28 × 10^0^	9.16 × 10^0^	1.47 × 10^2^	−1.50 × 10^1^	2.21 × 10^2^	1.97 × 10^2^	3.06 × 10^1^	1.92 × 10^−1^
40	Sulfur he-afluoride	Air	4.80 × 10^−2^	4.79 × 10^−2^	-	-	1.27 × 10^1^	1.24 × 10^1^	-	-
41	Sulfur o-ides	Air	-	-	-	-	-	-	-	-
42	Sulfur trio-ide	Air	-	-	-	-	5.00 × 10^0^	5.00 × 10^0^	-	-
43	Thallium	Water	-	-	-	-	-	-	-	-
44	Transformation. from forest (total)	Raw materials	1.79 × 10^0^	1.78 × 10^0^	8.19 × 10^0^	4.52 × 10^0^	3.28 × 10^1^	3.13 × 10^1^	1.33 × 10^0^	8.35 × 10^−3^
45	Transformation. from shrub (total)	Raw materials	3.45 × 10^−2^	3.17 × 10^−2^	-	-	-	3.39 × 10^−1^	-	-
46	Transformation. to forest (total)	Raw materials	−1.65 × 10^0^	−1.62 × 10^0^	-	−4.92 × 10^0^	−2.59 × 10^1^	−2.59 × 10^1^	−1.20 × 10^0^	−7.55 × 10^−3^
47	Transformation. to shrub (total)	Raw materials	−3.37 × 10^−2^	−3.09 × 10^−2^	-	-	-	−3.32 × 10^−1^	-	-
48	Vanadium	Air	2.39 × 10^−2^	2.38 × 10^−2^	-	-	-	-	-	-
49	Vanadium	Water	8.11 × 10^−1^	8.05 × 10^−1^	-	-	-	7.86 × 10^−1^	-	-
50	Water (total)	Water	−3.05 × 10^2^	−3.06 × 10^2^	−6.12 × 10^2^	−6.81 × 10^2^	−1.86 × 10^4^	−1.86 × 10^4^	−7.74 × 10^1^	−4.87 × 10^−1^
51	Water. cooling. unspecified natural origin (total)	Raw materials	3.35 × 10^0^	3.59 × 10^0^	5.51 × 10^1^	−4.78 × 10^0^	1.69 × 10^2^	1.65 × 10^2^	8.40 × 10^−1^	5.28 × 10^−3^
52	Water. lake (total)	Raw materials	-	-	-	-	1.18 × 10^0^	1.73 × 10^0^	-	-
53	Water. river (total)	Raw materials	1.19 × 10^−1^	1.50 × 10^−1^	6.08 × 10^0^	-	2.45 × 10^1^	2.57 × 10^1^	8.47 × 10^−1^	5.33 × 10^−3^
54	Water. turbine use. unspecified natural origin (total)	Raw materials	3.27 × 10^2^	3.04 × 10^2^	6.01 × 10^4^	−2.65 × 10^4^	2.77 × 10^4^	1.47 × 10^4^	9.28 × 10^2^	5.84 × 10^0^
55	Water. unspecified natural origin (total)	Raw materials	6.89 × 10^−1^	6.85 × 10^−1^	-	-	5.59 × 10^1^	5.59 × 10^1^	1.37 × 10^0^	8.62 × 10^−3^
56	Water. well (total)	Raw materials	1.83 × 10^−1^	1.96 × 10^−1^	-	-	5.81 × 10^0^	6.53 × 10^0^	1.44 × 10^−1^	9.06 × 10^−4^
57	Zinc	Air	2.15 × 10^−2^	2.12 × 10^−2^	-	-	3.74 × 10^0^	3.67 × 10^0^	8.14 × 10^−1^	5.12 × 10^−3^
58	Zinc	Water	2.95 × 10^0^	1.90 × 10^−1^	1.23 × 10^1^	1.21 × 10^1^	2.80 × 10^1^	2.71 × 10^1^	9.80 × 10^−1^	6.16 × 10^−3^
59	Zinc	Soil	-	-	-	-	-	-	-	-
60	Remaining substances	-	7.66 × 10^−1^	4.21 × 10^−1^	4.42 × 10^1^	1.06 × 10^1^	4.96 × 10^1^	5.00 × 10^1^	1.24 × 10^0^	7.80 × 10^−3^
	**Total**		**1.93 × 10^2^**	**1.14 × 10^2^**	**6.06 × 10^4^**	**−2.72 × 10^4^**	**1.05 × 10^4^**	**−2.66 × 10^3^**	**9.49 × 10^2^**	**5.97 × 10^0^**

**Table 7:** Grouping and weighting the consequences for the environment of the life cycle of the analyzed photovoltaic power plant, regarding their impact on the depletion of raw materials, taking into account the method of the post-used management of materials, components, and elements [unit: Pt].

No	Element of a Technical Object		Photovoltaic Power Plant	
	Waste Scenario		Landfill	Recycling
	Substance	Emission Area		
1	Aluminum	Raw materials	3.93 × 10^1^	−1.27 × 10^1^
2	Barite	Raw materials	-	−4.90 × 10^−3^
3	Chromium	Raw materials	7.44 × 10^−1^	7.64 × 10^−1^
4	Clay, bentonite	Raw materials	1.25 × 10^−2^	1.25 × 10^−2^
5	Clay, unspecified	Raw materials	2.82 × 10^−1^	2.26 × 10^−1^
6	Coal, hard	Raw materials	6.43 × 10^1^	2.09 × 10^1^
7	Cobalt	Raw materials	1.66 × 10^1^	1.66 × 10^1^
8	Copper	Raw materials	5.62 × 10^1^	5.62 × 10^1^
9	Copper, 0.99% in sulfide, Cu 0.36% and Mo 8.2 × 10^−3^% in crude ore	Raw materials	3.59 × 10^−1^	1.94 × 10^−2^
10	Copper, 1.18% in sulfide, Cu 0.39% and Mo 8.2 × 10^−3^% in crude ore	Raw materials	2.20 × 10^0^	2.05 × 10^−1^
11	Copper, 1.42% in sulfide, Cu 0.81% and Mo 8.2 × 10^−3^% in crude ore	Raw materials	5.28 × 10^−1^	2.84 × 10^−2^
12	Copper, 2.19% in sulfide, Cu 1.83% and Mo 8.2 × 10^−3^% in crude ore	Raw materials	2.89 × 10^0^	2.69 × 10^−1^
13	Gallium	Raw materials	-	3.28 × 10^−3^
14	Gas, natural/m^3^	Raw materials	3.31 × 10^2^	2.53 × 10^2^
15	Gold	Raw materials	5.05 × 10^0^	5.05 × 10^0^
16	Hafnium	Raw materials	-	3.89 × 10^−3^
17	Iron	Raw materials	5.48 × 10^1^	5.45 × 10^1^
18	Lead	Raw materials	4.96 × 10^0^	4.85 × 10^0^
19	Magnesium	Raw materials	3.11 × 10^1^	3.14 × 10^1^
20	Manganese	Raw materials	6.94 × 10^−1^	4.44 × 10^−1^
21	Molybdenum	Raw materials	1.47 × 10^0^	1.02 × 10^0^
22	Molybdenum, 0.010% in sulfide, Mo 8.2 × 10^−3^% and Cu 1.83% in crude ore	Raw materials	1.50 × 10^0^	7.67 × 10^−2^
23	Molybdenum, 0.014% in sulfide, Mo 8.2 × 10^−3^% and Cu 0.81% in crude ore	Raw materials	2.03 × 10^−1^	1.09 × 10^−2^
24	Molybdenum, 0.022% in sulfide, Mo 8.2 × 10^−3^% and Cu 0.36% in crude ore	Raw materials	8.88 × 10^−2^	−1.09 × 10^−2^
25	Molybdenum, 0.025% in sulfide, Mo 8.2 × 10^−3^% and Cu 0.39% in crude ore	Raw materials	7.43 × 10^−1^	4.01 × 10^−2^
26	Nickel	Raw materials	8.63 × 10^1^	8.63 × 10^1^
27	Nickel, 1.98% in silicates, 1.04% in crude ore	Raw materials	8.30 × 10^−1^	−3.49 × 10^−2^
28	Oil, crude	Raw materials	4.73 × 10^2^	1.32 × 10^2^
29	Palladium	Raw materials	2.08 × 10^−1^	2.08 × 10^−1^
30	Phosphorus	Raw materials	-	7.14 × 10^−3^
31	Platinum	Raw materials	1.59 × 10^−1^	1.59 × 10^−1^
32	Rhodium	Raw materials	-	2.78 × 10^−2^
33	Selenium	Raw materials	-	6.82 × 10^−3^
34	Silicon	Raw materials	5.87 × 10^1^	5.87 × 10^1^
35	Silver	Raw materials	1.07 × 10^1^	1.07 × 10^1^
36	Tin	Raw materials	1.15 × 10^1^	7.76 × 10^−2^
37	TiO2, 45–60% in Ilmenite	Raw materials	7.45 × 10−3	−3.11 × 10^−3^
38	Titanium	Raw materials	4.92 × 10^−2^	5.87 × 10^−2^
39	Uranium	Raw materials	3.95 × 10^−1^	1.70 × 10^−1^
40	Zinc	Raw materials	7.30 × 10^0^	7.30 × 10^0^
41	Remaining substances	-	6.89 × 10^−1^	3.82 × 10^−1^
**Total**			**1.26 × 10^3^**	**7.29 × 10^2^**

**Table 8:** Grouping and weighting the consequences of the life cycle of individual units of the analyzed photovoltaic power plant for the environment, regarding their impact on the depletion of raw materials, taking into account the method of post-used management of materials, components, and elements [unit: Pt].

No	Element of a Technical Object		Support Structure		Photovoltaic Panels		Inverter Station		Electrical Installation	
	Waste Scenario		Landfill	Recycling	Landfill	Recycling	Landfill	Recycling	Landfill	Recycling
	Impact Category	Emission Area								
1	Aluminum	Raw materials	4.26 × 10^−3^	4.13 × 10^−3^	3.08 × 10^1^	−1.44 × 10^1^	8.51 × 10^0^	1.70 × 10^0^	1.56 × 10^−2^	9.81 × 10^−5^
2	Barite	Raw materials	-	-	-	−4.90 × 10^−3^	-	-	-	-
3	Chromium	Raw materials	-	-	-	2.71 × 10^−2^	7.37 × 10^−1^	7.37 × 10^−1^	7.34 × 10^−3^	4.62 × 10^−5^
4	Clay, bentonite	Raw materials	1.25 × 10^−2^	1.25 × 10^−2^	-	-	-	-	-	-
5	Clay, unspecified	Raw materials	1.42 × 10^−2^	1.41 × 10^−2^	6.81 × 10^−2^	3.03 × 10^−2^	1.88 × 10^−1^	1.82 × 10^−1^	1.14 × 10^−2^	7.17 × 10^−5^
6	Coal, hard	Raw materials	9.22 × 10^0^	9.21 × 10^0^	2.97 × 10^1^	−7.32 × 10^0^	2.46 × 10^1^	1.90 × 10^1^	8.18 × 10^−2^	5.14 × 10^−3^
7	Cobalt	Raw materials	-	-	1.30 × 10^−1^	1.30 × 10^−1^	1.65 × 10^1^	1.65 × 10^1^	-	-
8	Copper	Raw materials	1.13 × 10^−2^	1.13 × 10^−2^	7.28 × 10^−1^	7.28 × 10^−1^	5.55 × 10^1^	5.55 × 10^1^	7.50 × 10^−3^	4.75 × 10^−3^
9	Copper, 0.99% in sulfide, Cu 0.36% and Mo 8.2 × 10^−3^% in crude ore	Raw materials	-	-	-	1.71 × 10^−2^	-	-	3.59 × 10^−1^	2.26 × 10^−3^
10	Copper, 1.18% in sulfide, Cu 0.39% and Mo 8.2 × 10^−3^% in crude ore	Raw materials	-	-	1.06 × 10^−1^	9.49 × 10^−2^	9.90 × 10^−2^	9.73 × 10^−2^	1.99 × 10^0^	1.25 × 10^−2^
11	Copper, 1.42% in sulfide, Cu 0.81% and Mo 8.2 × 10^−3^% in crude ore	Raw materials	-	-	-	2.51 × 10^−2^	-	-	5.28 × 10^−1^	3.32 × 10^−3^
12	Copper, 2.19% in sulfide, Cu 1.83% and Mo 8.2 × 10^−3^% in crude ore	Raw materials	-	-	1.40 × 10^−1^	1.25 × 10^−1^	1.30 × 10^−1^	1.28 × 10^−2^	2.62 × 10^0^	1.65 × 10^−2^
13	Gallium	Raw materials	-	-	-	3.28 × 10^−3^	-	-	-	-
14	Gas, natural/m^3^	Raw materials	3.91 × 10^0^	3.86 × 10^0^	8.97 × 10^1^	2.47 × 10^1^	1.33 × 10^2^	1.24 × 10^2^	4.34 × 10^0^	2.73 × 10^−2^
15	Gold	Raw materials	-	-	4.67 × 10^0^	4.67 × 10^0^	3.79 × 10^−1^	3.79 × 10^−1^	-	-
16	Hafnium	Raw materials	-	-	-	3.89 × 10^−3^	-	-	-	-
17	Iron	Raw materials	5.49 × 10^0^	5.48 × 10^0^	4.72 × 10^−1^	2.15 × 10^−1^	4.88 × 10^1^	4.88 × 10^1^	2.44 × 10^−2^	1.53 × 10^−4^
18	Lead	Raw materials	-	-	2.66 × 10^−1^	1.71 × 10^−1^	4.69 × 10^0^	4.68 × 10^0^	-	-
19	Magnesium	Raw materials	-	-	-	2.52 × 10^−1^	3.11 × 10	3.11 × 10^1^	-	-
20	Manganese	Raw materials	6.28 × 10^−2^	6.28 × 10^−3^	2.52 × 10^−1^	2.55 × 10^−3^	3.79 × 10^−1^	3.79 × 10^−1^	-	-
21	Molybdenum	Raw materials	8.86 × 10^−3^	8.36 × 10^−3^	3.50 × 10^−1^	1.68 × 10^−1^	8.70 × 10^−1^	8.43 × 10^−1^	2.41 × 10^−1^	1.52 × 10^−3^
22	Molybdenum, 0.010% in sulfide, Mo 8.2 × 10^−3^% and Cu 1.83% in crude ore	Raw materials	-	-	7.98 × 10^−2^	6.98 × 10^−2^	-	-	1.42 × 10^0^	8.93 × 10^−3^
23	Molybdenum, 0.014% in sulfide, Mo 8.2 × 10^−3^% and Cu 0.81% in crude ore	Raw materials	-	-	-	9.65 × 10^−3^	-	-	2.03 × 10^−1^	1.28 × 10^−3^
24	Molybdenum, 0.022% in sulfide, Mo 8.2 × 10^−3^% and Cu 0.36% in crude ore	Raw materials	-	-	7.93 × 10^−2^	−1.10 × 10^−2^	-	-	9.49 × 10^−3^	5.97 × 10^−5^
25	Molybdenum, 0.025% in sulfide, Mo 8.2 × 10^−3^% and Cu 0.39% in crude ore	Raw materials	-	-	-	3.54 × 10^−2^	-	-	7.43 × 10^−1^	4.67 × 10^−3^
26	Nickel	Raw materials	1.99 × 10^−2^	1.99 × 10^−2^	2.19 × 10^0^	2.19 × 10^0^	8.41 × 10^1^	8.41 × 10^1^	4.87 × 10^−3^	3.06 × 10^−5^
27	Nickel, 1.98% in silicates, 1.04% in crude ore	Raw materials	3.74 × 10^−3^	-	3.19 × 10^−1^	−3.81 × 10^−2^	-	-	5.07 × 10^−1^	3.19 × 10^−3^
28	Oil, crude	Raw materials	1.25 × 10**^1^**	1.21 × 10^1^	2.47 × 10^2^	−4.39 × 10^1^	0.78 × 10^2^	0.64 × 10^2^	5.38 × 10^0^	3.38 × 10^−2^
29	Palladium	Raw materials	-	-	2.08 × 10^−1^	2.08 × 10^−1^	-	-	-	-
30	Phosphorus	Raw materials	-	-	-	7.14 × 10^−3^	-	-	-	-
31	Platinum	Raw materials	-	-	1.59 × 10^−1^	1.59 × 10^−1^	-	-	-	-
32	Rhodium	Raw materials	-	-	-	2.78 × 10^−2^	-	-	-	-
33	Selenium	Raw materials	-	-	-	6.82 × 10^−3^	-	-	-	-
34	Silicon	Raw materials	-	-	2.05 × 10^−1^	2.05 × 10^−1^	5.85 × 10^1^	5.85 × 10^1^	-	-
35	Silver	Raw materials	-	-	7.22 × 10^0^	7.22 × 10^0^	3.51 × 10^0^	3.51 × 10^0^	-	-
36	Tin	Raw materials	-	-	-	5.30 - 10^−3^	-	-	1.15 × 10^1^	7.23 × 10^−2^
37	TiO2, 45–60% in Ilmenite	Raw materials	-	-	-	−3.16 × 10^−3^	-	-	7.45 × 10^−3^	4.69 × 10^−5^
38	Titanium	Raw materials	4.92 × 10^−2^	4.92 × 10^−2^	-	9.54 × 10^−3^	-	-	-	-
39	Uranium	Raw materials	-	-	1.48 × 10^−1^	−4.76 × 10^−2^	2.43 × 10^−1^	2.15 × 10^−1^	3.60 × 10^−3^	2.26 × 10^−5^
40	Zinc	Raw materials	-	-	4.74 × 10^−1^	4.72 × 10^−1^	6.83 × 10^0^	6.83 × 10^0^	-	-
41	Remaining substances	-	1.74 × 10^−2^	1.63 × 10^−2^	2.29 × 10^−1^	2.31 × 10^−3^	4.37 × 10^−1^	3.63 × 10^−1^	5.83 × 10^−3^	3.67 × 10^−5^
	**Total**		**3.13 × 10^1^**	**3.08 × 10^1^**	**4.66 × 10^2^**	**−2.78 × 10^1^**	**3.14 × 10^2^**	**2.79 × 10^2^**	**3.05 × 10^1^**	**1.92 × 10^−1^**

## Data Availability

The original contributions presented in this study are included in the article. Further inquiries can be directed to the corresponding author.
